# Clinical Relevance of Drug–Drug Interactions With Antibiotics as Listed in a National Medication Formulary: Results From Two Large Population‐Based Case‐Control Studies in Patients Aged 65–100 Years Using Linked English Primary Care and Hospital Data

**DOI:** 10.1002/cpt.2807

**Published:** 2022-12-16

**Authors:** Tjeerd Pieter van Staa, Munir Pirmohamed, Anita Sharma, Iain Buchan, Darren M. Ashcroft

**Affiliations:** ^1^ Centre for Health Informatics & Health Data Research UK North, Division of Informatics, Imaging and Data Science, School of Health Sciences, Faculty of Biology, Medicine and Health, Manchester Academic Health Science Centre The University of Manchester Manchester UK; ^2^ Centre for Drug Safety Science, Institute of Systems, Molecular and Integrative Biology University of Liverpool Liverpool UK; ^3^ Chadderton South Health Centre, Eaves Lane, Chadderton Oldham UK; ^4^ Institute of Population Health, NIHR Applied Research Collaboration North West Coast University of Liverpool Liverpool UK; ^5^ Centre for Pharmacoepidemiology and Drug Safety, National Institute for Health Research Greater Manchester Patient Safety Translational Research Centre, School of Health Sciences, Faculty of Biology, Medicine and Health The University of Manchester Manchester UK

## Abstract

This study evaluated drug–drug interactions (DDIs) between antibiotic and nonantibiotic drugs listed with warnings of severe outcomes in the British National Formulary based on adverse drug reaction (ADR) detectable with routine International Classification of Diseases, Tenth Revision coding. Data sources were Clinical Practice Research Databank GOLD and Aurum anonymized electronic health records from English general practices linked to hospital admission records. In propensity‐matched case‐control study, outcomes were ADR or emergency admissions. Analyzed were 121,546 ADR‐related admission cases matched to 638,238 controls. For most antibiotics, adjusted odds ratios (aORs) for ADR‐related hospital admission were large (aOR for trimethoprim 4.13; 95% confidence interval (CI), 3.97–4.30). Of the 51 DDIs evaluated for ADR‐related admissions, 38 DDIs (74.5%) had statistically increased aORs of concomitant exposure compared with nonexposure (mean aOR 3.96; range 1.59–11.42); for the 89 DDIs for emergency hospital admission, the results were 75 (84.3%) and mean aOR 2.40; range 1.43–4.17. Changing reference group to single antibiotic exposure reduced aORs for concomitant exposure by 76.5% and 83.0%, respectively. Medicines listed to cause nephrotoxicity substantially increased risks that were related to number of medicines (aOR was 2.55 (95% CI, 2.46–2.64) for current use of 1 and 10.44 (95% CI, 7.36–14.81) for 3 or more medicines). In conclusion, no evidence of substantial risk was found for multiple DDIs with antibiotics despite warnings of severe outcomes in a national formulary and flagging in electronic health record software. It is proposed that the evidence base for inclusion of DDIs in national formularies be strengthened and made publicly accessible and indiscriminate flagging, which compounds alert fatigue, be reduced.

**STUDY HIGHLIGHTS**

**WHAT IS THE CURRENT KNOWLEDGE ON THE TOPIC?**

National medicine formularies list large numbers of drug–drug interactions (DDIs).

**WHAT QUESTION DID THIS STUDY ADDRESS?**

This study evaluated DDIs between antibiotic and nonantibiotic drugs listed with warnings of severe outcomes in the British National Formulary based on adverse drug reaction (ADR) detectable with routine International Classification of Diseases, Tenth Revision coding.

**WHAT DOES THIS STUDY ADD TO OUR KNOWLEDGE?**

Of the 51 DDIs evaluated for ADR‐related admissions, 74.5% had statistically increased odds ratios of concomitant exposure of antibiotic and nonantibiotic compared with nonexposure. However, the differences were small when comparing concomitant to exposure to single antibiotic exposure (odds ratios reduced by on average 76.5%).

**HOW MIGHT THIS CHANGE CLINICAL PHARMACOLOGY OR TRANSLATIONAL SCIENCE?**

It is proposed that the evidence base for inclusion of DDIs in national formularies is strengthened and made publicly accessible and indiscriminate flagging in electronic health record systems be reduced.

Around 6.5% of hospital admissions are thought to be related to adverse drug reactions (ADRs), with drug–drug interactions (DDIs) accounting for 16.6% of the ADRs.^1^ A meta‐analysis of 81 studies reported that around 1 in 30 patients are exposed to preventable medication harm.^2^ A review of 13 studies found that the median DDI prevalence in hospital admissions was 1.1%,^3^ similar to the figure identified in our paper.^1^ However, there is limited evidence of which pairs of medicines increase DDI risks. A review of studies of elderly hospitalized patients by Hines *et al*.^4^ showed that there were only 17 studies of DDIs, with antibiotics implicated in 7 of these studies.

Electronic health record (EHR) software now frequently flags up warnings during consultations when a clinician prescribes listed interacting medicines. For example, EMIS (formerly known as Egton Medical Information Systems), which is the most frequently used EHR software in English primary care,^5^ bases its interaction flagging on Appendix One of the British National Formulary (BNF) and on the Summary of Product Characteristics or Drug Safety Updates by the UK regulatory authority (personal communication EMIS 2021). The BNF “… addresses the day‐to‐day prescribing information needs of healthcare professionals. Use of this resource throughout the health service helps to ensure that medicines are used safely, effectively, and appropriately.”^6^ BNF Appendix One is around 200 pages, listing DDIs that are classified according to level of severity and evidence (i.e., based on theoretical, study, or anecdotal evidence). For example, the BNF states that “… macrolides increases the concentration of digoxin …” (severe severity and anecdotal evidence).^7^ A high‐severity warning is automatically generated in the EMIS EHR when coprescribing these medicines. These represent potential DDIs, and the information on the absolute risks and frequencies of these DDIs and the patient groups at highest risk are generally not provided in formularies such as the BNF. Only a general warning is included that patients at increased risk of DDI include the elderly and those with impaired renal or hepatic function.^7^ As DDIs are frequently flagged to prescribers, such information about clinical relevance and patient groups at highest risks may be useful. The overall aim of this study was to evaluate in an elderly population the effects of DDIs between antibiotics and nonantibiotics on the risk of hospital admission (including ADR‐related and emergency admissions). This also included an evaluation whether concomitant exposure of antibiotic and nonantibiotic increased hospital admission risks compared with single exposure of these drugs.

## METHODS

### Data

Clinical Practice Research Databank (CPRD GOLD)^8^ and Aurum^9^ were the data sources. Both databases contain longitudinal, anonymized, patient‐level EHRs from general practices in the United Kingdom. Almost all UK residents are registered with a general practice, which typically provides most of the primary healthcare. The general practice is informed about a patient's urgent care attendances at the hospital, admissions or clinic visits. General practices can use one of several clinical information systems, of which EMIS is commonest. Vision software was commonly used in the past but has reduced substantially in recent years in England.^5^ The CPRD GOLD databases includes general practices that use the Vision EHR software system while Aurum practices use EMIS Web. Practices can change their EHR software although this will be reflected in the start and end of data collection for each practice. CPRD GOLD includes data on about 11.3 million patients^8^ and Aurum 19 million patients,^9^ although practices and patients may have contributed data for varying durations of time. These databases include the clinical diagnoses, medicines prescribed, vaccination history, lifestyle information, and clinical referrals, as well as patient's age, sex, ethnicity, smoking history, and body mass index. The patient‐level data from the general practices in England only has been linked through a trusted third party to hospital admission data (hospital episode statistics) using unique patient identifiers.^8^ The hospital data contained information on the date of hospital admission and the clinical diagnoses established at and during admission and coded using International Classification of Diseases, Tenth Revision (ICD‐10). Also, linked data were available, starting April 1 2007, for visits to emergency departments, including the visit day, but presenting diagnosis data was less complete for these visits. The general practices included in this study were from England, agreeing to record linkage. Patient‐level socioeconomic information was approximated from small area‐level Index of Multiple Deprivation (IMD) linked to the patient's residential postcode.^10^ Patient‐level IMD was aggregated into quintiles for the current analysis. GPs almost always prescribe medicines through the EHR. Most medicines for chronic conditions are prescribed at 2‐month intervals. Prescriptions were classified using the BNF sections. The rationale for using both CPRD GOLD and Aurum was to maximize statistical power in measuring the outcomes of interest.

### Study population

The overall study population consisted of patients aged 65–100 years at any time during the observation period. Follow‐up observation started on January 1, 2000, and ended on July 1, 2020, (CPRD GOLD) or September 1, 2020 (Aurum). Follow‐up of individual patients also considered their start date of registration with a general practice, prior history of registration in the practice of at least 3 years, time of reaching age 65 as well as end date due to moving away or death, and time of reaching age 101. The follow‐up of each patient was divided into 3‐month periods with risk factors such as presence of morbidity assessed at each of these time periods. Morbidity indicators included the QAdmission score, which estimates the risk of emergency hospital admission for patients aged 18–100 years in primary care.^11^ It is based on variables such as deprivation score, ethnicity, lifestyle variables (smoking, alcohol intake), and chronic diseases.^11^ Larger clusters of comorbidity were also identified using the k‐means method. Using 38 conditions,^12^ the number of clusters was increased stepwise until the number of patients in smaller clusters exceeded 5% of the size of the population. Patients were also classified at each 3 month period into frail yes/no groups (based on the QFrailty classification^11^). Patients with severe or moderate QFrailty were classified as frail (categories 1 and 2); those with mild and no frailty were classified as nonfrail (3 and 4). These morbidity indicators were used in the matching process.

Outcomes of interest included hospital admissions with ADR‐related codes and emergency hospital admission. Cases with an admission code for infection or peptic ulcer (indications for treatment including *Helicobacter pylori* infections) were excluded as these outcomes are also indications for treatment. For ADR‐related hospital admissions, we used a code list based on a systematic search and assessment of lists in 41 publications identifying ADRs from administrative data.^13^ This review classified codes according to level of likely causality based on the ICD‐10 code. The categories used in the current study included (i) ICD‐10 codes with phrase *induced by medication/drug*, (ii) ICD‐10 codes with phrase *induced by medication or other causes* or *poisoning by medication*, (iii) ADRs deemed to be very likely, or (iv) likely although the ICD‐10 code description does not refer to a drug.^13^ The primary outcomes were those with an admission code for ADRs. The secondary outcome was hospitalization with an ADR‐related code recorded at any time (i.e., codes at admission or discharge or during hospitalization). Such codes may include either ADRs that may not be fully determined at the time of admission but only get documented sometime into the admission following further investigation, or ADRs due to treatments given in the hospital. Emergency hospital admissions were defined as hospital admissions with a visit to the Accident & Emergency Department on the same day as the hospital admission, following the approach of Budnitz.^14^

The exposures of interest include DDIs with antibiotics using the following selection criteria: (i) listed in the BNF as severe (irrespective of level of evidence); (ii) listed in BNF tables with drugs that cause hepatotoxicity, nephrotoxicity, myelosuppression, prolonged QT interval or with neuromuscular blocking effects;^7^ or (iii) included in the review by Hines *et al*.^4^ of potentially harmful DDIs in the elderly. Up‐to‐date information on DDIs listed in the BNF can be found at https://bnf.nice.org.uk/interactions/#f. A total of 2,051 DDIs as listed in the BNF, and which involved antibiotics, were selected to begin with. The following exposure groups were defined: (i) no use (no prescribing of either of the interacting medicines in 90 days before index date); (ii) current concomitant use (both antibiotic and nonantibiotic prescribed in the 45 days prior to the index date); (iii) current single use of antibiotic (antibiotic prescribed in the 45 days before and no prescribing of the other medicine in the 90 days before); (iv) current single use of nonantibiotic; (v) recent single use of either antibiotic or nonantibiotic (one prescribed in the 45 days before and the other not in the 90 days before); (vi) recent concomitant use (both prescribed in 46–90 days before); and (vii) unclassified use (one current and the other past prescribed). In the analysis of antibiotic exposure (irrespective of other medicines), a past exposure group was also analyzed (i.e., last use in the 90–182 days before). A minimum number of cases and controls with concomitant exposure was applied (*n* = 25 for ADR‐related and *n* = 50 for emergency) as the list of DDIs included medicines rarely prescribed in primary care.

Cases were patients with a first hospital admission of interest during follow‐up. Cases were matched to up to six controls without hospital admission on the index date (hospital admission date of case). The objective of the matching was to closely match on the extent of morbidity based on disease (although not on treatments). Matching was done using propensity matching (using the QAdmission score) as well as matching by variables including age, sex, morbidity cluster, presence of frailty, practice coding level, and calendar time. Age and calendar time matching was done stepwise (age same year up to difference of up to 5 years; calendar time from within 3 months up to difference up to 5 years). For each practice, the mean level of coding was assessed for each general practice based on the average of nine inception cohorts of starters of medicines. The presence of a code for the indication of treatment was measured and then averaged across the practice. Matching was done separately for CPRD GOLD and Aurum and the risk‐set approach to control sampling was used (with control patients potentially included as controls for multiple cases, although only once for a particular case).

### Statistical analysis

The propensity matching procedure used a caliper (i.e., prespecified maximum difference) of 0.25 of the logit of the propensity score.^15^ Greedy nearest neighbor matching was used to select the control unit nearest to each treated unit. The Statistical Analysis System procedure PSMATCH (SAS Institute, Cary, NC) was used to conduct the matching. Conditional logistic regression was used to estimate the odds ratios (ORs) and 95% confidence intervals (CIs) for hospital admission. In the conditional logistic regression, the effects of different exposures were based on comparing each case with matched controls (estimating ORs and 95% CIs). Potential confounders in the regression models included IMD quintiles, smoking history and indicator of missingness, ethnicity (other ethnicity and missing ethnicity with White patients as reference), and individual morbidities (to further minimize residual confounding by underlying morbidity). The morbidities included atrial fibrillation, cancer, asthma or chronic obstructive pulmonary disease, congestive heart failure, cardiovascular disease, dementia, epilepsy, falls, learning disability, leg ulcer, chronic liver or renal disease or pancreatitis, Parkinson's disease, rheumatoid arthritis, chronic kidney disease, type 1 diabetes, type 2 diabetes, schizophrenia, dementia, and venous thromboembolism. The Charlson comorbidity index was also estimated as an indicator of overall level of morbidity.^16^ All analyses were performed using Statistical Analysis System software version 9.4.

## RESULTS

A total of 121,546 ADR‐related hospital admission cases was included in the analysis. They were propensity matched to 638,238 controls; cases and controls were well matched on age, sex, and comorbidity characteristics (as shown in **Table** **1**). The mean age of cases from CPRD Aurum was 78.6 years, compared with 78.5 years in one randomly selected control. Baseline characteristics for emergency hospital cases and controls are found in **Table** **S1**.

**Table 1:** Characteristics of ADR‐related hospital admission cases and propensity‐matched controls stratified by data source^a^

	Cases	CPRD GOLD		Cases	CPRD Aurum	
		Controls	One control per case		Controls	One control per case
	*N* = 13,856	*N* = 61,015	*N* = 13,856	*N* = 70,786	*N* = 379,319	*N* = 70,786
Sex women (%)	8,080 (58.3%)	36,804 (60.3%)	8,080 (58.3%)	40,426 (57.1%)	220,497 (58.1%)	40,426 (57.1%)
Age mean (SD)	78.5 (8.3)	77.4 (8.0)	78.4 (8.2)	78.6 (8.2)	78.1 (8.1)	78.5 (8.2)
Charlson score						
Very low (score 0)	3,421 (24.7%)	22,256 (36.5%)	4,105 (29.6%)	16,692 (23.6%)	117,083 (30.9%)	19,886 (28.1%)
Low (1,2)	5,001 (36.1%)	24,222 (39.7%)	5,553 (40.1%)	24,838 (35.1%)	147,679 (38.9%)	26,842 (37.9%)
Moderate (3,4)	3,539 (25.5%)	10,630 (17.4%)	2,886 (20.8%)	17,377 (24.5%)	77,058 (20.3%)	15,443 (21.8%)
High (5,6)	1,365 (9.9%)	3,072 (5%)	998 (7.2%)	8,185 (11.6%)	27,661 (7.3%)	6,103 (8.6%)
Very high (7+)	530 (3.8%)	835 (1.4%)	314 (2.3%)	3,694 (5.2%)	9,838 (2.6%)	2,512 (3.5%)
Risk score for hospital admissions mean (SD)	16.4 (11.4)	13.5 (9.2)	16.1 (11)	16.3 (11.7)	14.6 (10.2)	15.9 (11.3)
Risk score for mortality (mean)	9.1 (10.1)	6.8 (7.9)	8.7 (9.7)	10.1 (10.9)	8.6 (9.4)	9.7 (10.4)
Medical history						
Atrial fibrillation	1,806 (13.0%)	5,485 (9.0%)	1,857 (13.4%)	10,394 (14.7%)	50,570 (13.3%)	10,943 (15.5%)
Congestive heart failure	1,380 (10.0%)	3,186 (5.2%)	1,166 (8.4%)	8,530 (12.1%)	33,018 (8.7%)	7,577 (10.7%)
Cancer	807 (5.8%)	2,678 (4.4%)	908 (6.6%)	5,602 (7.9%)	28,085 (7.4%)	6,589 (9.3%)
Asthma/chronic obstructive lung disease	2,273 (16.4%)	8,683 (14.2%)	2,501 (18%)	12,811 (18.1%)	65,688 (17.3%)	13,702 (19.4%)
Cardiovascular disease	4,501 (32.5%)	16,404 (26.9%)	4,641 (33.5%)	23,867 (33.7%)	119,579 (31.5%)	24,568 (34.7%)
Diabetes mellitus type 2	3,145 (22.7%)	11,337 (18.6%)	2,975 (21.5%)	17,160 (24.2%)	82,250 (21.7%)	16,422 (23.2%)
Dementia	918 (6.6%)	3,012 (4.9%)	886 (6.4%)	4,064 (5.7%)	17,258 (4.5%)	3,516 (5.0%)

ADR, adverse drug reaction; CPRD, Clinical Practice Research Datalink.

^a^ Cases and controls were matched on several morbidity indicators.

As shown in **Table** **S2**, the incidence rate of ADR‐related hospital admission was strongly dependent upon the presence of frailty (and age). The rate was 5.54 per 1,000 person‐years in those with severe frailty and 0.93 in those without frailty. The rate was 1.78 per 1,000 person‐years in women and 1.93 in men.

**Table** **2** shows the adjusted ORs for ADR‐related hospital admissions for individual antibiotic types. For most antibiotics, a strong time response was found with highest ORs for current exposure and lowest for past exposure for different antibiotic types. For trimethoprim, the adjusted OR was 4.13 (95% CI, 3.97–4.30) for current and 1.39 (95% CI, 1.32–1.46) for past exposure. There were considerable differences in risk of ADR‐related hospital admission between different antibiotic types, (even with the same class).

**Table 2:** Adjusted ORs for ADR‐related hospital admissions stratified by current, recent, and past exposure for different antibiotic types

Antibiotic class	Antibiotic type	Count of currently exposed users	Current exposure	Recent exposure	Past exposure
			Adjusted OR (95% CI)	Adjusted OR (95% CI)	Adjusted OR (95% CI)
Cephalosporins and other beta‐lactams	Cefalexin	6,230	3.04 (2.86–3.23)	1.82 (1.67–1.98)	1.55 (1.45–1.66)
	Cefaclor	687	2.16 (1.78–2.61)	1.69 (1.34–2.13)	1.44 (1.20–1.73)
	Cefradine	593	2.70 (2.21–3.30)	2.30 (1.77–3 0)	1.56 (1.25–1.93)
	Cefadroxil	219	3.30 (2.41–4.53)	1.70 (1.13–2.57)	1.31 (0.92–1.86)
	Cefuroxime	85	3.58 (2.19–5.83)	1.40 (0.70–2.79)	1.82 (1.11–2.98)
Clindamycin and lincomycin	Clindamycin	194	3.65 (2.62–5.07)	1.70 (1.09–2.66)	1.50 (1.06–2.13)
Macrolides	Clarithromycin	5,002	2.61 (2.44–2.80)	1.44 (1.31–1.58)	1.32 (1.23–1.42)
	Erythromycin	3,279	2.36 (2.16–2.57)	1.50 (1.34–1.68)	1.37 (1.26–1.49)
	Azithromycin	794	1.83 (1.52–2.20)	1.30 (0.88–1.91)	0.98 (0.69–1.37)
Penicillins	Amoxicillin	23,458	2.03 (1.96–2.11)	1.27 (1.22–1.33)	1.15 (1.11–1.20)
	Flucloxacillin	9,610	2.55 (2.43–2.69)	1.67 (1.57–1.78)	1.38 (1.31–1.45)
	Co‐amoxiclav	5,548	3.44 (3.23–3.66)	1.65 (1.51–1.80)	1.42 (1.33–1.52)
	Penicillin	2,232	1.96 (1.76–2.18)	1.69 (1.44–1.98)	1.41 (1.25–1.59)
	Ampicillin	457	1.84 (1.44–2.35)	1.30 (0.95–1.78)	1.32 (1.05–1.65)
	Pivmecillinam	347	3.84 (3.01–4.91)	2.09 (1.50–2.91)	1.09 (0.81–1.49)
Quinolones	Ciprofloxacin	3,586	4.41 (4.09–4.76)	2.27 (2.05–2.52)	1.83 (1.69–1.98)
	Norfloxacin	144	3.17 (2.15–4.68)	1.90 (1.16–3.09)	1.69 (1.11–2.56)
	Ofloxacin	113	2.68 (1.72–4.19)	2.10 (1.13–3.92)	1.76 (1.12–2.76)
	Levofloxacin	111	3.56 (2.29–5.54)	1.93 (1.04–3.57)	1.93 (1.23–3.05)
Tetracyclines	Doxycycline	5,574	1.74 (1.62–1.87)	1.29 (1.18–1.42)	1.20 (1.12–1.30)
	Oxytetracycline	1,383	1.16 (0.99–1.37)	1.21 (0.98–1.50)	1.05 (0.89–1.25)
	Lymecycline	345	1.03 (0.74–1.43)	0.68 (0.37–1.27)	1.17 (0.76–1.80)
	Tetracycline	157	2.09 (1.39–3.14)	2.15 (1.27–3.67)	1.68 (1.15–2.46)
Sulphonamides and trimethoprim	Trimethoprim	14,226	4.13 (3.97–4.30)	1.63 (1.54–1.74)	1.39 (1.32–1.46)
	Co‐trimoxazole	126	2.22 (1.43–3.44)	1.64 (0.84–3.21)	3.43 (1.86–6.33)
Urinary tract infections	Nitrofurantoin	6,295	2.76 (2.60–2.94)	1.61 (1.47–1.76)	1.39 (1.29–1.49)
	Methenamine	114	1.99 (1.24–3.18)	1.51 (0.45–5.10)	0.42 (0.05–3.32)
Metronidazole, tinidazole, and ornidazole	Metronidazole	1,612	5.75 (5.15–6.42)	3.11 (2.71–3.58)	1.89 (1.69–2.11)
Some other antibacterials	Sodium fusidate	48	7.60 (4.03–14.34)	2.68 (0.96–7.46)	1.63 (0.69–3.81)

ADR, adverse drug reaction; CI, confidence interval; ORs, odds ratios.

Multiple mediation pairs listed as DDIs had increased ORs for ADR‐related hospital admission (**Table** **3** and **Table** **S3**). However, single exposure of one of the two drugs, particularly single exposure of antibiotics, also had increased ORs. For example, concomitant exposure of flucloxacillin and atorvastatin was associated with an adjusted OR of 2.58 (95% CI, 2.23–2.98) for ADR‐related admission compared with nonexposure, while this OR reduced to 1.00 (95% CI, 0.86–1.17) compared with single exposure of flucloxacillin. The analysis of the outcome of hospital admission with ADR codes at any time (rather than just admission code) showed similar results. Analyses of emergency admissions also found patterns of increased ORs of DDI compared with nonexposure but fewer when comparing to single antibiotic exposure (for flucloxacillin and atorvastatin, the adjusted ORs were 1.87 (95% CI, 1.77–1.98) and 0.94 (95% CI, 0.88–1.00), respectively).

**Table 3:** Concomitant and single exposure to antibiotics and potentially interacting drugs and adjusted ORs for ADR‐related and emergency hospital admissions

Antibiotic	Other drug	Counts of concomitant users	ADR‐related hospital admission			
			Admission record			Any record
			Concomitant compared with nonexposure	Concomitant compared with other drug single	Concomitant compared with antibiotic single	Concomitant compared with nonexposure
			Adjusted OR (95% CI)	Adjusted OR (95% CI)	Adjusted OR (95% CI)	Adjusted OR (95% CI)
Flucloxacillin	Paracetamol	1,895	3.21 (2.88–3.58)	2.24 (2.01–2.50)	1.25 (1.11–1.41)	2.59 (2.45–2.73)
Flucloxacillin	Atorvastatin	1,123	2.58 (2.23–2.98)	2.41 (2.09–2.79)	1.00 (0.86–1.17)	2.06 (1.92–2.22)
Doxycycline	Paracetamol	1,091	2.32 (2.00–2.70)	1.62 (1.39–1.88)	1.32 (1.11–1.56)	2.23 (2.09–2.38)
Doxycycline	Atorvastatin	910	1.60 (1.34–1.92)	1.51 (1.26–1.80)	0.91 (0.75–1.10)	1.72 (1.59–1.85)
Flucloxacillin	Simvastatin	395	2.33 (1.83–2.98)	2.23 (1.74–2.85)	0.92 (0.71–1.18)	2.05 (1.81–2.33)
Trimethoprim	Diclofenac	352	5.96 (4.71–7.55)	4.01 (3.16–5.10)	1.46 (1.15–1.86)	3.52 (3.07–4.04)
Erythromycin	Atorvastatin	279	2.26 (1.69–3.02)	2.13 (1.59–2.85)	0.94 (0.70–1.28)	1.81 (1.55–2.11)
Clarithromycin	Digoxin	274	3.62 (2.74–4.78)	2.81 (2.12–3.71)	1.39 (1.05–1.86)	2.94 (2.54–3.40)
Trimethoprim	Ibuprofen	203	4.96 (3.61–6.81)	3.85 (2.79–5.32)	1.21 (0.88–1.67)	3.86 (3.25–4.59)
Amoxicillin	Warfarin	199	1.97 (1.40–2.78)	1.75 (1.23–2.48)	0.98 (0.69–1.38)	1.94 (1.65–2.29)
Cefalexin	Diclofenac	191	5.05 (3.62–7.03)	3.38 (2.42–4.73)	1.68 (1.20–2.35)	3.09 (2.54–3.76)
Oxytetracycline	Paracetamol	191	1.97 (1.35–2.89)	1.37 (0.94–2.01)	1.81 (1.19–2.77)	1.53 (1.26–1.87)
Doxycycline	Simvastatin	161	1.59 (1.06–2.38)	1.54 (1.03–2.32)	0.92 (0.61–1.38)	2.06 (1.76–2.43)
Erythromycin	Digoxin	157	6.07 (4.17–8.83)	4.73 (3.25–6.90)	2.71 (1.84–3.98)	2.57 (2.11–3.13)
Trimethoprim	Naproxen	149	7.43 (5.19–10.62)	5.09 (3.53–7.33)	1.83 (1.28–2.62)	4.31 (3.56–5.22)
Erythromycin	Simvastatin	146	2.92 (1.98–4.32)	2.84 (1.92–4.19)	1.27 (0.85–1.89)	2.37 (1.93–2.91)
Oxytetracycline	Atorvastatin	137	1.35 (0.84–2.17)	1.27 (0.79–2.05)	1.19 (0.72–1.97)	1.20 (0.93–1.55)
Ciprofloxacin	Diclofenac	137	11.42 (7.62–17.11)	7.76 (5.16–11.65)	2.70 (1.79–4.08)	5.34 (4.21–6.76)
Cefalexin	Ibuprofen	137	3.15 (2.10–4.73)	2.44 (1.62–3.68)	1.04 (0.69–1.57)	3.28 (2.57–4.18)
Erythromycin	Citalopram	115	2.74 (1.78–4.22)	1.60 (1.04–2.47)	1.15 (0.74–1.79)	2.46 (1.99–3.04)
**Emergency hospital admission**						
Flucloxacillin	Paracetamol	13,736	2.23 (2.14–2.33)	1.75 (1.67–1.82)	1.10 (1.05–1.15)	
Doxycycline	Paracetamol	9,926	2.28 (2.17–2.39)	1.78 (1.70–1.87)	1.08 (1.02–1.14)	
Flucloxacillin	Atorvastatin	8,100	1.87 (1.77–1.98)	1.93 (1.83–2.05)	0.94 (0.88–1.00)	
Doxycycline	Atorvastatin	7,870	1.85 (1.74–1.96)	1.91 (1.81–2.03)	0.89 (0.84–0.95)	
Trimethoprim	Digoxin	4,691	2.02 (1.88–2.18)	1.92 (1.79–2.07)	0.90 (0.83–0.96)	
Flucloxacillin	Simvastatin	2,833	1.86 (1.70–2.04)	1.94 (1.77–2.13)	0.94 (0.85–1.03)	
Erythromycin	Atorvastatin	1,841	1.61 (1.43–1.81)	1.67 (1.48–1.88)	0.79 (0.70–0.90)	
Trimethoprim	Diclofenac	1,832	2.86 (2.56–3.19)	2.14 (1.91–2.39)	1.27 (1.14–1.42)	
Doxycycline	Simvastatin	1,734	1.92 (1.71–2.17)	2.01 (1.78–2.26)	0.94 (0.83–1.06)	
Clarithromycin	Digoxin	1,722	2.33 (2.07–2.61)	2.23 (1.98–2.50)	0.94 (0.83–1.06)	
Amoxicillin	Warfarin	1,599	1.85 (1.64–2.09)	1.78 (1.57–2.02)	0.91 (0.81–1.03)	
Oxytetracycline	Paracetamol	1,341	1.48 (1.28–1.71)	1.16 (1.00–1.34)	1.16 (0.98–1.36)	
Trimethoprim	Ibuprofen	1,313	3.57 (3.15–4.04)	2.53 (2.23–2.87)	1.60 (1.41–1.81)	
Erythromycin	Simvastatin	980	2.09 (1.79–2.44)	2.18 (1.87–2.55)	1.05 (0.90–1.23)	
Oxytetracycline	Atorvastatin	953	1.14 (0.94–1.37)	1.18 (0.98–1.43)	0.89 (0.73–1.09)	
Erythromycin	Digoxin	945	2.06 (1.76–2.40)	1.97 (1.69–2.31)	1.03 (0.88–1.21)	
Cefalexin	Diclofenac	942	2.65 (2.28–3.09)	1.98 (1.70–2.31)	1.32 (1.13–1.53)	
Erythromycin	Citalopram	932	2.45 (2.10–2.86)	1.78 (1.53–2.08)	1.22 (1.04–1.43)	
Trimethoprim	Naproxen	913	3.77 (3.25–4.38)	2.42 (2.08–2.81)	1.69 (1.45–1.96)	
Flucloxacillin	Warfarin	737	1.77 (1.48–2.12)	1.71 (1.42–2.04)	0.89 (0.74–1.07)	

ADR‐related and emergency hospital admissions selected based on BNF‐listed DDIs; top 20 for counts of concomitant medication users.

ADR, adverse drug reaction; BNF, British National Formulary; CI, confidence interval; DDIs, drug–drug interactions; ORs, odds ratios.

A total of 51 DDIs were evaluated for ADR‐related admissions and 89 for emergency hospital admission. Several DDIs were found to have adjusted ORs that were statistically significantly increased compared with nonexposure but not compared with single exposure of one of the medicines (**Table** **4**). Changing the reference group to single antibiotic exposure reduced the adjusted ORs for concomitant exposure by 76.5% and 83.0%, respectively (indicating presence of substantial effects associated with antibiotics rather than DDI effects).

**Table 4:** Effects of changing the reference group for concomitant exposure for hospital admissions with ADR‐related admission or with ADR record at any time during hospitalization and for emergency hospital admissions (based on the DDIs included in the main analyses)^a^

Type of hospital admission	Level of statistical significance \concomitant vs. nonexposure^b^	Level of statistical significance concomitant vs. single drug exposure^b^	No. DDI analyses (%)	No. patients with concomitant exposure (mean)	No. patients with single exposure nonantibiotic (mean)	No. patients with single exposure antibiotic (mean)	Mean OR concomitant vs. nonexposure (range ORs)	Mean OR concomitant vs. single antibiotic exposure (range ORs)	Reduction in ORs by changing reference to single antibiotic exposure^c^ (%)	Mean OR concomitant vs. sum of single antibiotic and nonantibiotic exposure^d^
ADR‐related										
Admission record	NS	NS	13 (25.5%)	42	22,921	4,832	1.69 (1.09–2.55)	1.01 (0.49–1.96)	98.5	0.81
	SIGN	NS	29 (56.9%)	163	17,213	6,687	3.42 (1.59–7.63)	1.25 (0.71–1.86)	82.2	0.94
	SIGN	SIGN	9 (17.6%)	552	30,531	7,843	5.68 (2.32–11.42)	1.82 (1.25–2.71)	65.6	1.28
	SIGN	NS or SIGN	38 (74.5%)	238	19,802	6,912	3.96 (1.59–11.42)	1.38 (0.71–2.71)	76.5	1.02
Any record	NS	NS	24 (26.7%)	72	71,227	10,846	1.43 (0.85–2.07)	0.84 (0.33–1.46)	100	0.77
	SIGN	NS	48 (53.3%)	249	36,982	18,954	2.67 (1.41–5.03)	1.17 (0.77–1.79)	84.2	0.92
	SIGN	SIGN	18 (20.0%)	1,110	68,824	20,900	3.65 (1.72–7.24)	1.61 (0.82–3.37)	63.4	1.07
	SIGN	NS or SIGN	66 (73.3%)	481	45,574	19,479	2.93 (1.41–7.24)	1.29 (0.77–3.70)	76.5	0.96
Emergency	NS	NS	14 (15.7%)	225	241,322	25,530	1.23 (0.70–1.73)	0.82 (0.54–1.26)	100	0.78
	SIGN	NS	58 (65.2%)	319	75,750	39,580	2.24 (1.43–3.23)	1.09 (0.78–1.61)	89.0	0.91
	SIGN	SIGN	17 (19.1%)	2,929	206,377	47,129	2.92 (1.61–4.17)	1.39 (0.79–2.07)	69.3	0.97
	SIGN	NS or SIGN	75 (84.3%	936	106,592	41,363	2.40 (1.43–4.17)	1.16 (0.78–2.07)	83.0	0.92

ADR, adverse drug reaction; DDIs, drug–drug interactions; OR, odds ratio.

^a^ NS, not statistically significant; SIGN, statistically significant.

^b^ Concomitant exposure compared with sum of effects of the single exposures of antibiotic and nonantibiotic (beta of concomitant exposure minus sum of betas of single exposures in model with nonexposure as reference).

^c^ Main analyses were restricted to DDIs with at least 25 ADR‐related hospital admissions in patients with concomitant exposure (at least 50 for emergency hospital admissions).

^d^ Reduction in ORs of concomitant exposure by changing reference from nonexposure to single antibiotic exposure (%).

**Figure 1:**
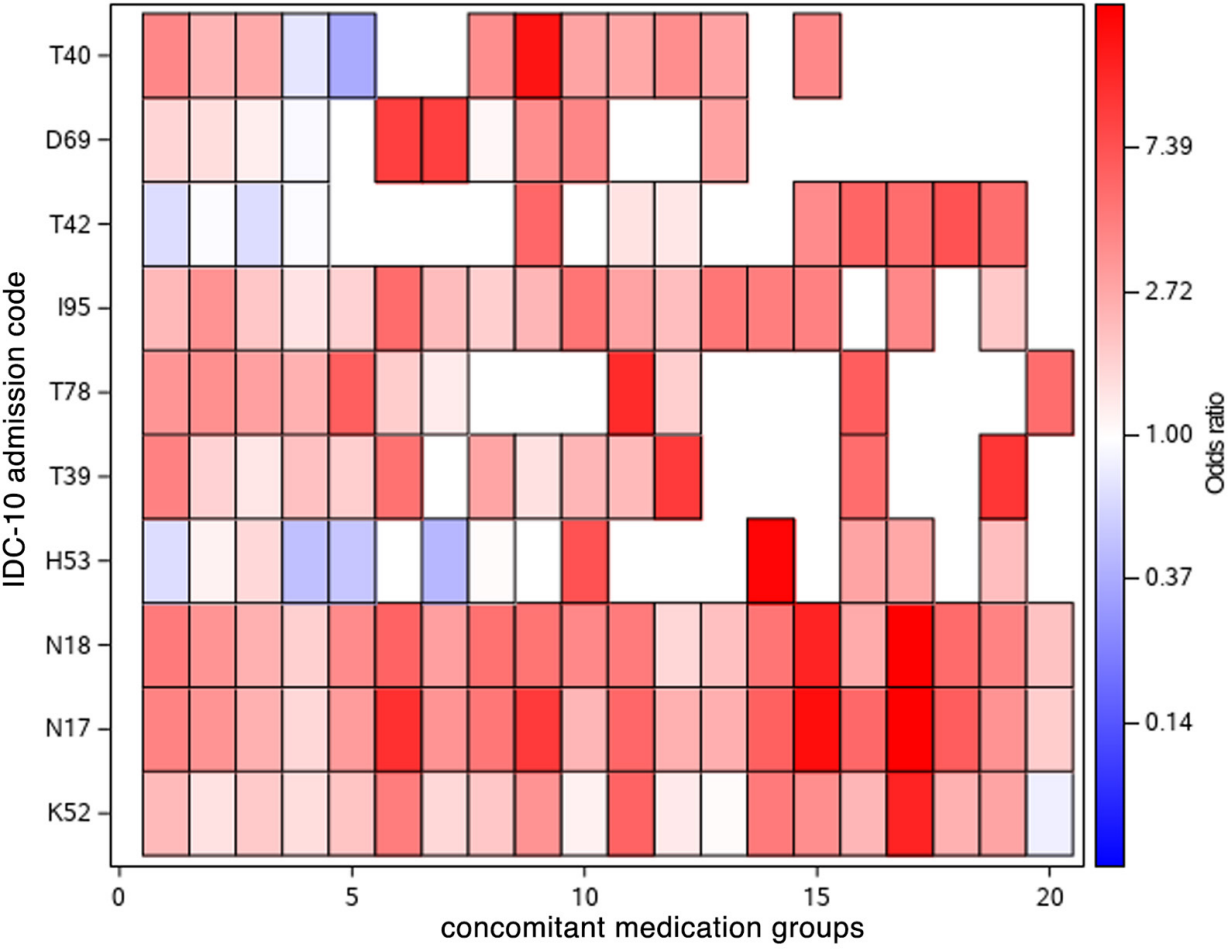
Heatmap of adjusted ORs of ADR‐related hospital admission stratified by ICD‐10 hospital admission codes (most frequent concomitant medication users and statistically significant ORs for concomitant exposure compared with nonuse). X variables for concomitant medication groups: 1, flucloxacillin‐paracetamol; 2, flucloxacillin‐atorvastatin; 3, doxycycline‐paracetamol; 4, doxycycline‐atorvastatin; 5, flucloxacillin‐simvastatin; 6, trimethoprim‐diclofenac; 7, erythromycin‐atorvastatin; 8, clarithromycin‐digoxin; 9, trimethoprim‐ibuprofen; 10, amoxicillin‐warfarin; 11, cefalexin‐diclofenac; 12, oxytetracycline‐paracetamol; 13, doxycycline‐simvastatin; 14, erythromycin‐digoxin; 15, trimethoprim‐naproxen; 16, erythromycin‐simvastatin; 17, ciprofloxacin‐diclofenac; 18, cefalexin‐ibuprofen; 19, erythromycin‐citalopram; 20, flucloxacillin‐warfarin. Y variable: ICD‐10 admission code: K52, other and unspecified noninfective gastroenteritis and colitis; N17, acute kidney failure; N18, chronic kidney disease; H53, visual disturbances; T39, poisoning by, adverse effect of, and underdosing of nonopioid analgesics, antipyretics, and antirheumatics; T78, adverse effects, not elsewhere classified; I95, hypotension; T42, poisoning by, adverse effect of, and underdosing of antiepileptic, sedative‐hypnotic, and antiparkinsonism drugs; D69, purpura and other hemorrhagic conditions; T40, poisoning by, adverse effect of, and underdosing of narcotics and psychodysleptics (hallucinogens). ADR, adverse drug reaction; ICD‐10, International Classification of Diseases, Tenth Revision; ORs, odds ratios.

**Figure** **1** displays a heatmap of adjusted ORs stratified by specific ICD‐10 hospital admission codes and concomitant exposure groups (top 20 in count of concomitant users). Admissions for acute kidney failure and chronic kidney disease were substantially increased with concomitant exposure of macrolides and digoxin. **Figure** **S1** also shows the results for comparisons of single antibiotic exposure (instead of concomitant exposure) to nonuse. Similar patterns of acute kidney failure and chronic kidney disease were found for single antibiotic and concomitant exposure; the results for other ICD‐10 codes were less clear due to smaller numbers. **Figure** **S2** displays a heatmap for emergency hospital admissions.

The analysis including the DDIs as reported by Hines *et al*. showed similar findings of generally increased risks of concomitant exposure compared with nonexposure which reduced substantially when comparing to single antibiotic exposure (**Table** **5**). Only concomitant exposure of macrolides and digoxin showed an increased OR in ADR‐related hospital admission compared with single exposure of macrolides (adjusted OR 1.59 (95% CI, 1.80–1.98)).

**Table 5:** Concomitant and single exposure to antibiotics and potentially interacting drugs and adjusted ORs for ADR‐related and emergency hospital admissions (selected based on DDIs reported in a review of epidemiological studies by Hines^4^)

Type of hospital admission	Antibiotic	Other drug	Counts of concomitant users	Concomitant compared with nonexposure	Concomitant compared with other drug single	Concomitant compared with antibiotic single
				Adj OR (95% CI)	Adj OR (95% CI)	Adj OR (95% CI)
ADR‐related hospital admission	Any antibiotic	Warfarin	5,162	3.20 (2.98–3.44)	2.82 (2.62–3.05)	1.11 (1.03–1.19)
	Ciprofloxacin	Aminophylline	36	1.75 (0.73–4.23)	1.37 (0.56–3.34)	0.40 (0.16–0.96)
	Any antibiotic	Sulphonylureas	4,661	3.25 (3.03–3.50)	2.74 (2.54–2.97)	1.13 (1.05–1.21)
	Macrolides	Digoxin	491	3.85 (3.11–4.77)	3.00 (2.42–3.71)	1.59 (1.28–1.98)
	Macrolides	Calcium blocker	1,984	2.83 (2.54–3.16)	2.73 (2.45–3.05)	1.20 (1.06–1.36)
Emergency admission	Any antibiotic	Warfarin	34,098	2.21 (2.15–2.28)	2.09 (2.03–2.16)	1.00 (0.97–1.03)
	Ciprofloxacin	Aminophylline	189	4.60 (3.35–6.33)	2.83 (2.05–3.90)	1.72 (1.25–2.37)
	Any antibiotic	Sulphonylureas	25,063	2.45 (2.38–2.54)	2.21 (2.13–2.28)	1.11 (1.08–1.15)
	Macrolides	Digoxin	2,983	2.14 (1.96–2.34)	2.05 (1.87–2.24)	0.95 (0.87–1.04)
	Co‐trimoxazole	Angiotensin receptor blocker	120	1.21 (0.75–1.95)	1.21 (0.75–1.96)	0.67 (0.40–1.12)
	Macrolides	Calcium channel blocker	14,996	2.37 (2.28–2.47)	2.35 (2.26–2.45)	1.07 (1.02–1.12)

Adj, adjusted; ADR, adverse drug reaction; CI, confidence interval; DDIs, drug–drug interactions; ORs, odds ratios.

As shown in **Table** **6**, medicines that have been listed in the BNF to cause nephrotoxicity were found to have substantially increased risks during current exposure, which dropped to lower levels during past exposure. Risks were related to number of medicines, although there was no indication of a statistical interaction (adjusted OR was 2.55 (95% CI, 2.46–2.64) for current use of one nephrotic medicine, while it was 10.44 (95% CI, 7.36–14.81) for 3 or more medicines). There was no indication of reductions in ORs over calendar time.

**Table 6:** Adjusted ORs for ADR‐related hospital admission for renal failure stratified by current, recent, and past exposure and number of medicines^a^

Stratum	Number of medicines	Count of currently exposed users	Current exposure	Recent exposure	Past exposure
			Adjusted OR (95% CI)	Adjusted OR (95% CI)	Adjusted OR (95% CI)
All	1	26,082	2.55 (2.46–2.64)	1.42 (1.34–1.5)	1.20 (1.15–1.26)
	2	2,424	6.00 (5.49–6.55)	1.49 (1.18–1.88)	1.18 (0.99–1.41)
	3+	144	10.44 (7.36–14.81)	3.19 (1.25–8.11)	1.95 (1.04–3.66)
2000–2004	1	2,783	2.11 (1.90–2.34)	1.39 (1.19–1.64)	1.21 (1.04–1.40)
	2+	273	5.28 (4.07–6.86)	1.02 (0.51–2.04)	1.57 (1.00–2.48)
2005–2009	1	6,094	2.38 (2.21–2.56)	1.63 (1.46–1.82)	1.29 (1.17–1.42)
	2+	690	6.38 (5.36–7.60)	1.69 (1.13–2.53)	1.62 (1.18–2.22)
2010–2014	1	7,075	2.91 (2.73–3.10)	1.43 (1.29–1.59)	1.22 (1.12–1.34)
	2+	752	6.76 (5.75–7.96)	1.40 (0.90–2.19)	1.14 (0.82–1.59)
2015+	1	6,146	2.60 (2.44–2.78)	1.29 (1.16–1.45)	1.10 (1.00–1.21)
	2+	556	6.29 (5.23–7.56)	1.85 (1.09–3.13)	0.84 (0.54–1.31)

ADR, adverse drug reaction; CI, confidence interval; ORs, odds ratios.

^a^ Aceclofenac, aciclovir, amikacin, amphotericin, azithromycin, cefaclor, cefadroxil, cefalexin, cefixime, cefotaxime, cefradine, ceftazidime, ceftriaxone, cefuroxime, ciclosporin, clarithromycin, colistimethate, dexketoprofen, diclofenac, diflunisal, digoxin, etoricoxib, erythtromycin, flurbiprofen, gentamicin, ibuprofen, indometacin, ketoprofen, meloxicam, methotrexate, naproxen, neomycin, penicillamine, piroxicam, sulindac, tacrolimus, tiaprofenic, tobramycin, tolfenamic, trimethoprim, valganciclovir, vancomycin, zoledronic acid, or zuclopenthixol.

As shown in **Table** **S4** and **S5**, sensitivity analyses were conducted using a time window for current exposure of 21 days (instead of 45 days). Adjusted ORs for ADR‐related hospital admission with concomitant exposure compared with nonexposure were generally higher, although the comparisons with single antibiotic exposure as reference group showed a similar result with a longer time window.

## DISCUSSION

The evaluation of a large number of concomitant exposures of antibiotics and nonantibiotics, with warnings for severe DDIs in a national formulary, were found to have no increased risks when compared with single exposures of these drugs. Exposure to antibiotics, irrespective of comedications, was associated with increased risks of hospital admissions; risks varied between antibiotic types. Medicines listed in the formulary to cause nephrotoxicity substantially increased risks which were related to the number of these medicines.

Both the BNF and related website (https://bnf.nice.org.uk) do not routinely publish the evidence for including DDI warnings, other than brief classifications. We were also unable to find reasons why DDIs were selected for EHR flagging (including the alerts in the United Kingdom's most widely used primary care software, EMIS), or what the effectiveness is of such flagging. The EMIS website states that “comprehensive, point‐of‐care guidance on quality, safety, and cost is authored by our clinical experts from national sources, including NICE [National Institute for Health and Care Excellence], MHR [Medicines and Healthcare products Regulatory Agency], pharmacist‐led IT‐based intervention to reduce clinically important medication errors, NHSE [National Health Service England], RCGP [Royal College of General Practitioners], and PHE [Public Health England]” but the evidence is not publicly provided.^17^ Previous research has found substantive differences in the content of DDI listings. A study from Sweden evaluated the potential difference between commonly used tools used to identify DDIs including Micromedex, Lexicomp, DRUID, Swedish Physician's Desk reference, and INTERCheck. It found that the prevalence of DDIs strongly depended on what source was used to classify DDIs.^18^ A study comparing the BNF, Thesaurus, and Micromedex found considerable variation in the DDIs included as well as variability in categorization of severity and clinical advice given.^19^ These inconsistencies in DDI listings may possibly indicate a lack of systematically collected evidence on the impact of DDIs and susceptible patient groups in usual clinical practice. There is a need for large‐scale DDI analytics using platforms that enable distributed queries of linked data from health systems such as OpenSAFELY (www.opensafely.org) embedded in Combined Intelligence for Population Health Action (CIPHA) (www.cipha.nhs.uk) or the European Health Data & Evidence Network (www.ehden.eu). These platforms have provided substantial, prompt intelligence in COVID‐19 responses.^20^, ^21^, ^22^ These large‐scale DDI analytics should be combined with systemic approaches in assessing biological plausibility of findings.

Clinical decision support that flag DDIs in EHRs have been found to have little or moderate effects on improving patient outcomes. Relevant reviews, although based on limited source evidence, found that the effectiveness of such systems relied heavily on the support provided alongside the flagging.^23^, ^24^ A Cochrane review of randomized trials and observational studies reported moderate improvements in the process of care, with the effectiveness improved by reminders for a response from the clinician and by providing an explanation of the reminder's content or advice.^25^ A workgroup on the usability of DDI CDS identified seven core characteristics. These included information on the clinical characteristics (such as the potential adverse clinical outcomes and incidence), contextual information and modifying factors (such as patient‐specific risk factors), recommended actions (such as alternative treatment, dosage reductions or monitoring), and strength of evidence.^26^ An interesting question is whether DDI alerts could be implemented selectively, which would reduce alert burden (e.g., for specific exposure characteristics, clinicians with specific training or specific institutions).^26^ Although DDI alerts are now widely implemented, there is only sparse evidence on the cost‐effectiveness of these systems.^23^ A recent review developed recommendations for improving DDI CDS include individualized and practical recommendations, need to improve alert specificity and consideration of human factors principles in the implementation.^27^ However, we were unable to find any studies of whether the (cost‐)effectiveness of the DDI flagging in UK primary care and user experiences were evaluated despite its wide implementation and what criteria were used in activating DDI flagging (although a study is ongoing to evaluate the effectiveness of OptimiseRx (https://www.nottingham.ac.uk/research/groups/medicinesafetyeffectivehealthcare/protect‐study/index.aspx)). In addition, accurate knowledge databases are needed to produce relevant DDI alerts,^27^ which would make the underlying evidence more transparent.

Seven studies have reported on DDIs with antibiotics and medicines such as calcium channel blockers, digoxin and sulphonylureas.^4^ These studies were population‐based using healthcare databases.^28^, ^29^, ^30^, ^31^, ^32^, ^33^, ^34^, ^35^ However, a major limitation of these studies may be that all were nested within users of one of the medicines. There was therefore no evaluation of the independent effects of single exposure to the other medicine and no assessment of whether the individual effects were modified by combined exposure. Also, most of the DDIs as listed in formularies such as the BNF have not been based on evidence from large population‐based studies with details on magnitude of DDI effect, incidence, and predictors. There is a need that the evidence base for inclusion of DDIs in national formularies is strengthened.

This study has several limitations that should be considered when interpreting the findings. Firstly, this study provided associations but not causal estimates. Residual confounding is possible, and the randomization that could disambiguate this would be infeasible. However, propensity matching by multiple variables was done to minimize confounding. Comparisons of different classes of antibiotics showed substantial differences in risk despite broadly comparable indications for treatment. A second limitation is that we used a broad set of clinical conditions to define ADR‐related outcomes, as based on a systematic literature search.^13^ But the measurement of more specific outcomes for each DDI based on biological mechanisms may have resulted in higher ORs,^36^ although the study lacked statistical power to detect rarer DDIs. The use of a broad outcome set was preferred in this study given the interest in evaluating a large number of DDIs and lack of information on specific outcomes for each DDI. A related limitation is that DDIs were limited to events that resulted in hospital admission. DDIs such as a prolonged QT interval were missed as they are usually asymptomatic or result in sudden cardiac death with most patients not reaching the hospital. Another limitation was that multiple statistical tests were conducted without probabilistic adjustment for multiple testing. The key finding, however, was robust to changing the reference group to current exposure, with a confirmatory reduction in risk—this was not part of a series of tests. A further limitation was that exposure data were based on prescription data and not on plasma levels. Not all patients will have adhered to the recommended treatments, which may have resulted in an underestimate of effect.

In conclusion, ADR‐related hospital admissions frequently occur, particularly in frail elderly. No evidence of substantial risk was found for multiple DDIs with antibiotics despite warnings of severe outcomes in a national formulary and automated flagging, which compounds alert fatigue. Medicines listed as causing nephrotoxicity had substantive risks of hospital admission. We suggest that publicly available evidence should be provided before including DDIs into national formularies and EHR prescribing alerts. Further research should implement large‐scale DDI analytics combined with systematic biological plausibility assessment to routinely evaluate risks.

## FUNDING

This study was supported by funding from the National Institute for Health and Care Research (Cluster randomized trial to improve antibiotic prescribing in primary care: individualized knowledge support during consultation for general practitioners and patients: Grant number NIHR130581) and Health Data Research UK (Better Care Northern Partnership, Better antibiotic prescribing in frail elderly people with polypharmacy: learning from practice and nudging prescribers into better practices BetterRx). DMA is funded by the National Institute for Health and Care Research through the Greater Manchester Patient Safety Translational Research Centre (NIHR Greater Manchester PSTRC, Grant number: PSTRC‐2016‐003). IB is funded by NIHR NW Coast Applied Research Collaboration.

## CONFLICTS OF INTEREST

I.B. is Chief Data Scientist Advisor for AstraZeneca. M.P. has received partnership funding for the following: Medical Research Council (MRC) Clinical Pharmacology Training Scheme (cofunded by MRC and Roche, Union Chimique Belge (UCB) Pharma, Eli Lilly, and Novartis); and a PhD studentship jointly funded by Engineering and Physical Sciences Research Council and Astra Zeneca. He has also received unrestricted educational grant support for the UK Pharmacogenetics and Stratified Medicine Network from Bristol‐Myers Squibb. He has developed a human leukocyte antigen genotyping panel with MC Diagnostics but does not benefit financially from this. None of these sources of funding were used for this article. All other authors declared no competing interests for this work.

## AUTHOR CONTRIBUTIONS

T.P.v.S., M.P., A.S., I.B., and D.M.A. wrote the manuscript. T.P.v.S., I.B., and D.M.A. designed the research. T.P.v.S. and A.S. performed the research. T.P.v.S. analyzed the data.

## Supporting information


Appendix S1
