# Supplementary material for: Clinical Relevance of Drug–Drug Interactions With Antibiotics as Listed in a National Medication Formulary: Results From Two Large Population‐Based Case‐Control Studies in Patients Aged 65–100 Years Using Linked English Primary Care and Hospital Data
Source: Clin Pharmacol Ther. 2022 Dec 16;113(2):423–34. doi: 10.1002/cpt.2807 (PMC10107602; doi:10.1002/cpt.2807)

**Table S1: Characteristics of cases with emergency hospital admission and propensity-matched controls stratified by data source^#^**

|  |  | CPRD GOLD | |  | CPRD Aurum | |
| --- | --- | --- | --- | --- | --- | --- |
|  | Cases | Controls | One control per case | Cases | Controls | One control per case |
|  | N=107058 | N=485668 | N=107058 | N=514041 | N=2813741 | N=514041 |
| Sex women (%) | 61292 (57.3%) | 283365 (58.3%) | 61292 (57.3%) | 287998 (56%) | 1593604 (56.6%) | 287998 (56%) |
| Age years mean (SD) | 79 (8.4) | 77.8 (8.2) | 78.8 (8.4) | 78.9 (8.3) | 78.5 (8.2) | 78.8 (8.3) |
| *Charlson score* | |  |  |  |  |  |
| Very low (score 0) | 29458 (27.5%) | 174952 (36%) | 32131 (30%) | 136659 (26.6%) | 876367 (31.1%) | 148363 (28.9%) |
| Low (1,2) | 41276 (38.6%) | 191057 (39.3%) | 42062 (39.3%) | 192109 (37.4%) | 1081561 (38.4%) | 194306 (37.8%) |
| Moderate (3,4) | 24840 (23.2%) | 89912 (18.5%) | 23379 (21.8%) | 120066 (23.4%) | 597590 (21.2%) | 115547 (22.5%) |
| High (5,6) | 8585 (8%) | 23855 (4.9%) | 7316 (6.8%) | 46681 (9.1%) | 197732 (7%) | 41523 (8.1%) |
| Very high (7+) | 2899 (2.7%) | 5892 (1.2%) | 2170 (2%) | 18526 (3.6%) | 60491 (2.1%) | 14302 (2.8%) |
| Risk score for hospital admissions mean (SD) | 16.2 (11.1) | 13.6 (9.1) | 15.8 (10.7) | 15.8 (11.1) | 14.5 (9.9) | 15.5 (10.8) |
| Risk score for mortality (mean) | 9 (9.8) | 6.9 (7.9) | 8.7 (9.5) | 9.8 (10.4) | 8.6 (9.1) | 9.5 (10.1) |
| *Medical history* | |  |  |  |  |  |
| Atrial fibrillation | 14193 (13.3%) | 45724 (9.4%) | 14174 (13.2%) | 71705 (13.9%) | 346268 (12.3%) | 71208 (13.9%) |
| Congestive heart failure | 6953 (6.5%) | 18770 (3.9%) | 6714 (6.3%) | 37571 (7.3%) | 161500 (5.7%) | 36300 (7.1%) |
| Cancer | 5869 (5.5%) | 18908 (3.9%) | 6162 (5.8%) | 38843 (7.6%) | 184847 (6.6%) | 41217 (8.0%) |
| Asthma / chronic obstructive lung disease | 20529 (19.2%) | 77447 (15.9%) | 20458 (19.1%) | 103860 (20.2%) | 506128 (18.0%) | 101537 (19.8%) |
| Cardiovascular disease | 32097 (30.0%) | 118927 (24.5%) | 32306 (30.2%) | 154762 (30.1%) | 782569 (27.8%) | 155879 (30.3%) |
| Diabetes mellitus type 2 | 18909 (17.7%) | 73485 (15.1%) | 18860 (17.6%) | 98557 (19.2%) | 509327 (18.1%) | 97898 (19.0%) |
| Dementia | 8879 (8.3%) | 28805 (5.9%) | 8124 (7.6%) | 36582 (7.1%) | 148865 (5.3%) | 29203 (5.7%) |

**^#^**cases and controls were matched on several morbidity indicators

**Table S2: Incidence rate of ADR-related hospital admission stratified by age, sex and frailty**

| Characteristics |  | Cases  (N=84642) | Person-time | Rate^#^ |
| --- | --- | --- | --- | --- |
|  |  |  |  |  |
| Sex | men | 48506 | 25182890 | 1.93 |
|  | women | 36136 | 20275940 | 1.78 |
|  |  |  |  |  |
| Age | 65-74 | 30198 | 23773362 | 1.27 |
|  | .75-84 | 32492 | 15214943 | 2.14 |
|  | 85+ | 21952 | 6470525 | 3.39 |
|  |  |  |  |  |
| Frailty (according to Qfrailty score) | None | 17488 | 18866333 | 0.93 |
|  | Minor | 44964 | 21491114 | 2.09 |
|  | Moderate | 16128 | 3984097 | 4.05 |
|  | Severe | 6062 | 1093859 | 5.54 |
|  |  |  |  |  |

^#^number of cases per 1000 person-years

**Table S3: Concomitant and single exposure to antibiotics and potentially interacting drugs and adjusted ORs for ADR-related and emergency hospital admissions (outside top 20 for counts of concomitant medication users)**

|  |  |  | ADR-related hospital admission | | | | | |
| --- | --- | --- | --- | --- | --- | --- | --- | --- |
|  |  |  |  | Admission record | | |  | Any record |
| Antibiotic | Other drug | Counts of concomitant users | Concomitant compared to non-exposure | | Concomitant compared to other drug single | Concomitant compared to antibiotic single | | Concomitant compared to non-exposure |
|  |  |  | Adj OR (95% CI) | | Adj OR (95% CI) | Adj OR (95% CI) | | Adj OR (95% CI) |
| flucloxacillin | warfarin | 111 | 1.80 (1.11-2.92) | | 1.56 (0.96-2.54) | 0.71 (0.44-1.15) | | 1.88 (1.48-2.40) |
| erythromycin | diltiazem | 96 | 2.83 (1.70-4.71) | | 2.60 (1.56-4.33) | 1.21 (0.72-2.03) | | 2.06 (1.60-2.64) |
| flucloxacillin | carbamazepine | 74 | 2.43 (1.41-4.19) | | 1.71 (0.98-2.97) | 0.96 (0.55-1.65) | | 2.24 (1.69-2.97) |
| ciprofloxacin | ibuprofen | 71 | 5.00 (2.91-8.60) | | 3.89 (2.25-6.71) | 1.13 (0.66-1.96) | | 3.10 (2.26-4.25) |
| azithromycin | digoxin | 70 | 1.54 (0.80-2.93) | | 1.20 (0.63-2.29) | 0.81 (0.41-1.59) | | 1.41 (1.01-1.98) |
| trimethoprim | meloxicam | 64 | 4.59 (2.63-8.02) | | 3.25 (1.84-5.75) | 1.12 (0.64-1.97) | | 3.58 (2.51-5.11) |
| lymecycline | atorvastatin | 63 | 1.09 (0.52-2.28) | | 1.02 (0.49-2.15) | 1.04 (0.46-2.38) | | 1.28 (0.91-1.79) |
| cefalexin | naproxen | 51 | 6.06 (3.24-11.34) | | 4.12 (2.19-7.74) | 2.02 (1.08-3.80) | | 4.07 (2.93-5.65) |
| co-amoxiclav | warfarin | 45 | 4.44 (2.21-8.92) | | 3.93 (1.96-7.91) | 1.30 (0.65-2.62) | | 3.18 (2.29-4.40) |
| flucloxacillin | fluconazole | 42 | 4.56 (2.31-8.98) | | 1.91 (0.95-3.85) | 1.80 (0.91-3.56) | | 3.51 (2.43-5.07) |
| erythromycin | buprenorphine | 40 | 3.63 (1.78-7.38) | | 2.29 (1.12-4.68) | 1.55 (0.76-3.17) | | 2.95 (2.12-4.11) |
| trimethoprim | ketoprofen | 40 | 4.80 (2.38-9.69) | | 3.83 (1.86-7.89) | 1.17 (0.58-2.37) | | 2.93 (1.96-4.40) |
| oxytetracycline | simvastatin | 39 | 1.91 (0.79-4.60) | | 1.87 (0.78-4.50) | 1.67 (0.68-4.10) | | 1.26 (0.85-1.87) |
| erythromycin | warfarin | 38 | 2.26 (1.11-4.63) | | 2.02 (0.98-4.14) | 0.97 (0.47-2.00) | | 3.24 (2.20-4.78) |
| ciprofloxacin | naproxen | 34 | 7.63 (3.71-15.7) | | 5.16 (2.50-10.65) | 1.76 (0.85-3.63) | | 3.23 (2.15-4.85) |
| lymecycline | paracetamol | 34 | 2.02 (0.82-5.00) | | 1.41 (0.57-3.48) | 1.96 (0.74-5.20) | | 1.16 (0.77-1.77) |
| doxycycline | carbamazepine | 33 | 2.49 (1.09-5.67) | | 1.78 (0.78-4.08) | 1.44 (0.63-3.28) | | 2.35 (1.67-3.30) |
| amoxicillin | methotrexate | 33 | 1.31 (0.52-3.32) | | 1.67 (0.64-4.38) | 0.65 (0.26-1.65) | | 1.59 (1.03-2.45) |
| flucloxacillin | fluvastatin | 32 | 1.80 (0.74-4.40) | | 1.84 (0.74-4.57) | 0.71 (0.29-1.74) | | 1.64 (0.86-3.15) |
| erythromycin | tolterodine | 32 | 2.01 (0.87-4.65) | | 1.45 (0.63-3.35) | 0.86 (0.37-1.99) | | 1.68 (1.07-2.63) |
| cefaclor | diclofenac | 31 | 3.70 (1.55-8.81) | | 2.48 (1.04-5.92) | 1.73 (0.71-4.22) | | 2.37 (1.25-4.48) |
| clarithromycin | colchicine | 30 | 4.24 (1.80-9.97) | | 2.08 (0.88-4.94) | 1.63 (0.69-3.85) | | 3.40 (2.23-5.20) |
| cefalexin | meloxicam | 29 | 5.57 (2.34-13.25) | | 3.98 (1.66-9.54) | 1.86 (0.78-4.43) | | 3.24 (1.79-5.87) |
| doxycycline | fluconazole | 28 | 2.55 (0.95-6.84) | | 1.02 (0.37-2.77) | 1.47 (0.55-3.95) | | 3.66 (2.58-5.18) |
| erythromycin | lercanidipine | 28 | 1.22 (0.43-3.42) | | 1.17 (0.41-3.30) | 0.52 (0.18-1.46) | | 2.14 (1.36-3.38) |
| ciprofloxacin | phenytoin | 27 | 5.39 (2.14-13.59) | | 3.95 (1.56-10.01) | 1.24 (0.49-3.13) | | 2.98 (1.68-5.29) |
| erythromycin | fentanyl | 26 | 1.16 (0.38-3.52) | | 0.53 (0.17-1.60) | 0.49 (0.16-1.49) | | 2.48 (1.65-3.73) |
| erythromycin | hydroxyzine | 26 | 2.80 (1.10-7.12) | | 1.38 (0.54-3.54) | 1.19 (0.47-3.04) | | 3.18 (1.89-5.35) |
| erythromycin | quinine | 26 | 2.16 (0.82-5.72) | | 1.47 (0.55-3.93) | 0.92 (0.34-2.43) | | 2.70 (1.79-4.07) |
| flucloxacillin | sulfasalazine | 26 | 2.99 (1.22-7.30) | | 3.09 (1.25-7.64) | 1.18 (0.48-2.89) | | 2.50 (1.60-3.91) |
| erythromycin | verapamil | 26 | 1.88 (0.75-4.75) | | 1.64 (0.65-4.17) | 0.80 (0.32-2.04) | | 2.36 (1.45-3.86) |
|  |  |  |  | |  |  | |  |
|  |  |  | Emergency hospital admission | | | | |  |
| erythromycin | diltiazem | 619 | 1.86 (1.52-2.26) | | 1.64 (1.34-2.00) | 0.92 (0.75-1.13) | |  |
| cefalexin | ibuprofen | 604 | 3.11 (2.57-3.76) | | 2.19 (1.81-2.65) | 1.55 (1.28-1.88) | |  |
| ciprofloxacin | diclofenac | 601 | 3.82 (3.18-4.58) | | 2.86 (2.38-3.43) | 1.43 (1.19-1.72) | |  |
| flucloxacillin | carbamazepine | 551 | 2.34 (1.91-2.87) | | 1.74 (1.42-2.14) | 1.18 (0.96-1.44) | |  |
| lymecycline | atorvastatin | 536 | 0.91 (0.69-1.19) | | 0.94 (0.72-1.24) | 0.98 (0.73-1.32) | |  |
| oxytetracycline | simvastatin | 442 | 1.14 (0.87-1.49) | | 1.20 (0.92-1.57) | 0.90 (0.68-1.18) | |  |
| lymecycline | paracetamol | 414 | 1.15 (0.86-1.55) | | 0.90 (0.67-1.21) | 1.26 (0.92-1.73) | |  |
| doxycycline | carbamazepine | 378 | 2.17 (1.69-2.79) | | 1.61 (1.25-2.07) | 1.06 (0.82-1.36) | |  |
| azithromycin | digoxin | 378 | 1.43 (1.09-1.88) | | 1.38 (1.05-1.81) | 0.85 (0.65-1.13) | |  |
| co-amoxiclav | warfarin | 373 | 2.51 (1.97-3.19) | | 2.41 (1.89-3.07) | 0.93 (0.73-1.18) | |  |
| ciprofloxacin | ibuprofen | 333 | 2.57 (1.99-3.30) | | 1.80 (1.40-2.31) | 0.95 (0.73-1.22) | |  |
| erythromycin | buprenorphine | 328 | 2.49 (1.91-3.25) | | 1.58 (1.20-2.06) | 1.24 (0.95-1.63) | |  |
| cefalexin | naproxen | 314 | 3.35 (2.59-4.34) | | 2.14 (1.65-2.78) | 1.66 (1.28-2.16) | |  |
| doxycycline | fluconazole | 298 | 3.64 (2.80-4.73) | | 1.82 (1.39-2.38) | 1.78 (1.36-2.31) | |  |
| flucloxacillin | fluconazole | 289 | 3.50 (2.67-4.60) | | 1.74 (1.31-2.30) | 1.76 (1.34-2.32) | |  |
| amoxicillin | methotrexate | 283 | 1.80 (1.34-2.43) | | 1.60 (1.18-2.18) | 0.89 (0.66-1.19) | |  |
| trimethoprim | meloxicam | 277 | 2.73 (2.05-3.63) | | 2.20 (1.64-2.94) | 1.22 (0.91-1.62) | |  |
| trimethoprim | ketoprofen | 240 | 2.06 (1.49-2.85) | | 1.73 (1.24-2.42) | 0.92 (0.66-1.27) | |  |
| erythromycin | quinine | 235 | 2.68 (1.98-3.62) | | 2.20 (1.62-2.98) | 1.34 (0.99-1.81) | |  |
| clarithromycin | risperidone | 225 | 1.93 (1.39-2.69) | | 1.41 (1.01-1.97) | 0.78 (0.56-1.09) | |  |
| erythromycin | fentanyl | 223 | 2.96 (2.16-4.05) | | 1.78 (1.30-2.45) | 1.48 (1.08-2.03) | |  |
| ciprofloxacin | naproxen | 220 | 2.93 (2.15-4.00) | | 1.86 (1.37-2.54) | 1.09 (0.80-1.49) | |  |
| erythromycin | tolterodine | 218 | 1.59 (1.10-2.29) | | 1.28 (0.89-1.85) | 0.79 (0.55-1.14) | |  |
| erythromycin | warfarin | 217 | 2.45 (1.79-3.36) | | 2.37 (1.73-3.25) | 1.23 (0.90-1.69) | |  |
| erythromycin | lercanidipine | 215 | 2.29 (1.66-3.17) | | 2.25 (1.63-3.12) | 1.15 (0.83-1.59) | |  |
| erythromycin | solifenacin | 206 | 2.35 (1.70-3.25) | | 1.90 (1.38-2.63) | 1.18 (0.85-1.63) | |  |
| flucloxacillin | sulfasalazine | 175 | 1.92 (1.31-2.80) | | 1.82 (1.24-2.67) | 0.96 (0.66-1.41) | |  |
| clarithromycin | colchicine | 166 | 2.48 (1.72-3.58) | | 1.98 (1.37-2.87) | 1.00 (0.69-1.45) | |  |
| erythromycin | carbamazepine | 157 | 2.03 (1.36-3.05) | | 1.51 (1.01-2.27) | 1.01 (0.68-1.52) | |  |
| tetracycline | paracetamol | 157 | 2.48 (1.68-3.66) | | 1.94 (1.32-2.86) | 1.41 (0.92-2.18) | |  |
| doxycycline | sulfasalazine | 146 | 1.84 (1.19-2.85) | | 1.75 (1.13-2.71) | 0.90 (0.58-1.39) | |  |
| erythromycin | verapamil | 137 | 2.18 (1.46-3.25) | | 1.95 (1.31-2.92) | 1.09 (0.73-1.63) | |  |
| erythromycin | quetiapine | 136 | 1.59 (1.04-2.44) | | 1.23 (0.80-1.88) | 0.79 (0.52-1.22) | |  |
| lymecycline | simvastatin | 132 | 0.69 (0.39-1.21) | | 0.73 (0.41-1.28) | 0.74 (0.41-1.31) | |  |
| erythromycin | sildenafil | 124 | 1.34 (0.82-2.19) | | 1.53 (0.93-2.51) | 0.67 (0.41-1.10) | |  |
| trimethoprim | aciclovir | 120 | 3.22 (2.14-4.87) | | 1.74 (1.14-2.65) | 1.44 (0.95-2.17) | |  |
| cefalexin | ketoprofen | 118 | 1.76 (1.10-2.81) | | 1.49 (0.93-2.40) | 0.87 (0.54-1.39) | |  |
| erythromycin | hydroxyzine | 116 | 2.18 (1.40-3.39) | | 1.70 (1.09-2.66) | 1.09 (0.70-1.70) | |  |
| cefalexin | meloxicam | 116 | 2.57 (1.62-4.07) | | 2.07 (1.30-3.29) | 1.27 (0.80-2.02) | |  |
| flucloxacillin | methotrexate | 115 | 2.58 (1.67-4.00) | | 2.36 (1.52-3.68) | 1.30 (0.84-2.02) | |  |
| erythromycin | risperidone | 109 | 3.06 (1.95-4.80) | | 2.25 (1.43-3.53) | 1.53 (0.97-2.41) | |  |
| erythromycin | venlafaxine | 109 | 1.94 (1.20-3.16) | | 1.44 (0.89-2.35) | 0.97 (0.60-1.58) | |  |
| doxycycline | methotrexate | 107 | 1.67 (1.03-2.70) | | 1.48 (0.91-2.42) | 0.81 (0.50-1.31) | |  |
| minocycline | paracetamol | 104 | 1.77 (1.07-2.96) | | 1.39 (0.83-2.31) | 1.58 (0.90-2.77) | |  |
| doxycycline | rosuvastatin | 95 | 1.90 (1.16-3.10) | | 1.93 (1.17-3.16) | 0.92 (0.57-1.51) | |  |
| flucloxacillin | rosuvastatin | 95 | 1.83 (1.07-3.15) | | 1.85 (1.07-3.19) | 0.92 (0.54-1.59) | |  |
| ciprofloxacin | phenytoin | 94 | 2.17 (1.35-3.51) | | 1.79 (1.11-2.89) | 0.81 (0.50-1.30) | |  |
| minocycline | atorvastatin | 93 | 0.85 (0.44-1.63) | | 0.88 (0.46-1.70) | 0.70 (0.35-1.40) | |  |
| tetracycline | atorvastatin | 93 | 2.27 (1.34-3.83) | | 2.36 (1.40-3.98) | 1.30 (0.75-2.26) | |  |
| cefaclor | diclofenac | 93 | 2.28 (1.39-3.74) | | 1.71 (1.04-2.79) | 1.00 (0.60-1.65) | |  |
| ciprofloxacin | meloxicam | 88 | 2.63 (1.56-4.45) | | 2.13 (1.25-3.60) | 0.98 (0.58-1.66) | |  |
| erythromycin | fluconazole | 86 | 2.23 (1.33-3.75) | | 1.10 (0.65-1.85) | 1.12 (0.67-1.88) | |  |
| erythromycin | colchicine | 83 | 2.45 (1.45-4.14) | | 1.96 (1.16-3.31) | 1.23 (0.73-2.07) | |  |
| cefradine | diclofenac | 80 | 1.92 (1.09-3.40) | | 1.44 (0.81-2.54) | 1.07 (0.60-1.91) | |  |
| cefalexin | methotrexate | 80 | 1.79 (1.02-3.13) | | 1.61 (0.91-2.83) | 0.89 (0.50-1.55) | |  |
| co-amoxiclav | methotrexate | 80 | 2.99 (1.78-5.05) | | 2.69 (1.59-4.56) | 1.11 (0.66-1.86) | |  |
| oxytetracycline | carbamazepine | 79 | 1.32 (0.73-2.40) | | 0.99 (0.54-1.79) | 1.05 (0.58-1.91) | |  |
| phenoxymethylpenicillin | warfarin | 78 | 2.69 (1.59-4.55) | | 2.61 (1.54-4.41) | 1.59 (0.94-2.70) | |  |
| flucloxacillin | fluvastatin | 76 | 1.07 (0.56-2.03) | | 1.12 (0.58-2.14) | 0.54 (0.28-1.02) | |  |
| flucloxacillin | pravastatin | 73 | 1.73 (0.96-3.11) | | 1.70 (0.94-3.08) | 0.87 (0.48-1.56) | |  |
| trimethoprim | methotrexate | 66 | 1.26 (0.65-2.43) | | 1.12 (0.58-2.16) | 0.56 (0.29-1.08) | |  |
| doxycycline | itraconazole | 63 | 3.16 (1.79-5.57) | | 1.97 (1.09-3.55) | 1.54 (0.87-2.72) | |  |
| erythromycin | haloperidol | 59 | 3.23 (1.75-5.96) | | 1.93 (1.04-3.57) | 1.61 (0.87-2.99) | |  |
| erythromycin | escitalopram | 58 | 2.53 (1.36-4.71) | | 1.99 (1.07-3.71) | 1.26 (0.68-2.36) | |  |
| trimethoprim | etodolac | 57 | 3.18 (1.74-5.79) | | 2.77 (1.50-5.10) | 1.42 (0.78-2.58) | |  |
| penicillin | warfarin | 57 | 2.00 (1.04-3.87) | | 1.95 (1.01-3.78) | 1.02 (0.52-2.02) | |  |
| erythromycin | methylprednisolone | 56 | 1.62 (0.82-3.20) | | 1.38 (0.70-2.72) | 0.81 (0.41-1.60) | |  |
| demeclocycline | paracetamol | 56 | 1.68 (0.85-3.34) | | 1.32 (0.66-2.61) | 0.85 (0.39-1.87) | |  |
| doxycycline | pravastatin | 56 | 1.36 (0.71-2.59) | | 1.33 (0.69-2.56) | 0.66 (0.35-1.26) | |  |
| cefalexin | aciclovir | 53 | 4.17 (2.27-7.66) | | 2.27 (1.23-4.20) | 2.07 (1.12-3.80) | |  |

**Table S4: Concomitant and single exposure to antibiotics and potentially interacting drugs and adjusted ORs for ADR-related hospital admission using a time-window for current exposure of 21 days**

| Antibiotic | Other drug | Counts of concomitant users | Concomitant compared to non-exposure | Concomitant compared to other drug single | Concomitant compared to antibiotic single |
| --- | --- | --- | --- | --- | --- |
|  |  |  | Adjusted OR | Adjusted OR | Adjusted OR |
| flucloxacillin | paracetamol | 702 | 3.79 (3.19-4.52) | 2.51 (2.11-2.99) | 1.31 (1.08-1.59) |
| flucloxacillin | atorvastatin | 401 | 2.82 (2.22-3.57) | 2.53 (2.00-3.21) | 0.97 (0.76-1.24) |
| doxycycline | paracetamol | 400 | 3.06 (2.43-3.85) | 2.01 (1.60-2.53) | 1.36 (1.06-1.76) |
| doxycycline | atorvastatin | 348 | 2.28 (1.75-2.97) | 2.07 (1.59-2.71) | 1.02 (0.77-1.36) |
| flucloxacillin | simvastatin | 159 | 2.30 (1.57-3.37) | 2.05 (1.39-3.00) | 0.80 (0.55-1.18) |
| trimethoprim | diclofenac | 143 | 8.01 (5.54-11.59) | 5.11 (3.52-7.43) | 1.32 (0.91-1.91) |
| erythromycin | atorvastatin | 104 | 2.32 (1.45-3.70) | 2.10 (1.31-3.36) | 0.80 (0.50-1.31) |
| oxytetracycline | paracetamol | 75 | 1.85 (1.01-3.37) | 1.22 (0.67-2.22) | 1.66 (0.86-3.18) |
| amoxicillin | warfarin | 72 | 2.21 (1.29-3.80) | 2.00 (1.15-3.46) | 0.88 (0.51-1.52) |
| cefalexin | diclofenac | 69 | 13.71 (7.47-25.17) | 8.78 (4.77-16.15) | 3.75 (2.03-6.92) |
| oxytetracycline | atorvastatin | 67 | 0.99 (0.45-2.14) | 0.90 (0.41-1.95) | 0.85 (0.38-1.90) |
| trimethoprim | naproxen | 67 | 10.77 (6.20-18.71) | 6.96 (3.97-12.19) | 1.79 (1.03-3.12) |
| doxycycline | simvastatin | 67 | 1.92 (1.06-3.49) | 1.79 (0.99-3.26) | 0.88 (0.48-1.60) |
| trimethoprim | ibuprofen | 66 | 7.16 (4.14-12.39) | 5.03 (2.89-8.74) | 1.18 (0.68-2.05) |
| cefalexin | ibuprofen | 57 | 4.84 (2.68-8.75) | 3.39 (1.87-6.16) | 1.32 (0.73-2.39) |
| erythromycin | simvastatin | 49 | 3.07 (1.57-6.01) | 2.84 (1.45-5.55) | 1.11 (0.56-2.19) |
| erythromycin | citalopram | 44 | 2.84 (1.42-5.66) | 1.58 (0.79-3.16) | 0.98 (0.49-1.97) |
| ciprofloxacin | diclofenac | 37 | 9.31 (4.40-19.72) | 5.93 (2.79-12.58) | 1.56 (0.73-3.33) |
| flucloxacillin | warfarin | 34 | 2.41 (1.02-5.70) | 2.03 (0.85-4.83) | 0.84 (0.35-2.00) |
| lymecycline | atorvastatin | 31 | 0.73 (0.22-2.47) | 0.66 (0.20-2.25) | 0.60 (0.16-2.20) |
| erythromycin | diltiazem | 31 | 2.26 (0.86-5.94) | 2.03 (0.77-5.34) | 0.80 (0.30-2.12) |
| ciprofloxacin | ibuprofen | 27 | 9.97 (4.15-23.97) | 7.00 (2.90-16.88) | 1.62 (0.67-3.92) |
| flucloxacillin | carbamazepine | 26 | 3.89 (1.62-9.30) | 2.67 (1.11-6.44) | 1.36 (0.57-3.27) |
| trimethoprim | meloxicam | 25 | 10.1 (4.30-23.71) | 7.59 (3.19-18.06) | 1.67 (0.71-3.92) |

**Table S5: Effects of changing the reference group for concomitant exposure for hospital admissions with ADR-related hospital admissions (based on the DDIs included in the main analyses£)**

| Type of hospital admission | Level of statistical significance \concomitant versus non-exposure^#^ | Level of statistical significance concomitant versus single drug exposure^#^ | N DDI analyses (%) | N patients with concomitant exposure (mean) | N patients with single exposure non-antibiotic (mean) | N patients with single exposure antibiotic (mean) | Mean OR concomitant versus non-exposure  (range ORs) | Mean OR concomitant versus single antibiotic exposure  (range ORs) | Reduction in ORs by changing reference to single antibiotic exposure* (%) | Mean OR concomitant versus sum of single antibiotic and non-antibiotic exposure^&^ |
| --- | --- | --- | --- | --- | --- | --- | --- | --- | --- | --- |
| Admission record | NS | NS | 3 (12.5%) | 43 | 34994 | 839 | 1.32 (0.99-2.26) | 0.75 (0.60-0.85) |  | 0.68 |
|  | SIGN | NS | 17 (70.8%) | 102 | 16963 | 4405 | 4.55 (2.84-10.10) | 1.18 (0.80-.67) | 89.3 | 0.89 |
|  | SIGN | SIGN | 4 (16.7%) | 310 | 26872 | 4515 | 7.83 (7.28-13.71) | 2.05 (1.31-3.75) | 65.0 | 1.33 |
|  | SIGN | NS or SIGN | 21 (87.5%) | 142 | 18851 | 4426 | 5.17 (3.07-13.71) | 1.34 (0.80-3.75) | 82.1 | 0.97 |

^£^Main analyses were restricted to DDIs with at least 25 ADR-related hospital admissions in patients with concomitant exposure

^#^NS=not statistically significant; SIGN=statistically significant

*reduction in ORs of concomitant exposure by changing reference from non-exposure to single antibiotic exposure (%)

^&^concomitant exposure compared to sum of effects of the single exposures of antibiotic and non-antibiotic (beta of concomitant exposure minus sum of betas of single exposures in model with non-exposure as reference)

**Figure S1: Heatmap of adjusted ORs of ADR-related hospital admission stratified by ICD10 hospital admission codes (most frequent concomitant medication users and statistically significant ORs concomitant and single antibiotic exposure compared to non-use)**

Legends to Figure S1

X variables concomitant medication groups

1=flucloxacillin-paracetamol

2=flucloxacillin-atorvastatin

3=doxycycline-paracetamol

4=doxycycline-atorvastatin

5=flucloxacillin-simvastatin

6=trimethoprim-diclofenac

7=erythromycin-atorvastatin

8=clarithromycin-digoxin

9=trimethoprim-ibuprofen

10=amoxicillin-warfarin

11=cefalexin-diclofenac

12=oxytetracycline-paracetamol

13=doxycycline-simvastatin

14=erythromycin-digoxin

15=trimethoprim-naproxen

16=erythromycin-simvastatin

17=ciprofloxacin-diclofenac

18=cefalexin-ibuprofen

19=erythromycin-citalopram

20=flucloxacillin-warfarin

Y variable: ICD10 admission code

K52=Other and unspecified noninfective gastroenteritis and colitis

N17=Acute kidney failure

N18=Chronic kidney disease (CKD)

H53=Visual disturbances

T39=Poisoning by, adverse effect of and underdosing of nonopioid analgesics, antipyretics and antirheumatics

T78=Adverse effects, not elsewhere classified

I95=Hypotension

T42=Poisoning by, adverse effect of and underdosing of antiepileptic, sedative- hypnotic and antiparkinsonism drugs

D69=Purpura and other haemorrhagic conditions

T40=Poisoning by, adverse effect of and underdosing of narcotics and psychodysleptics [hallucinogens]

**Figure S1**

| Concomitant exposure compared to non-use | Single antibiotic exposure compared to non-use |
| --- | --- |
| 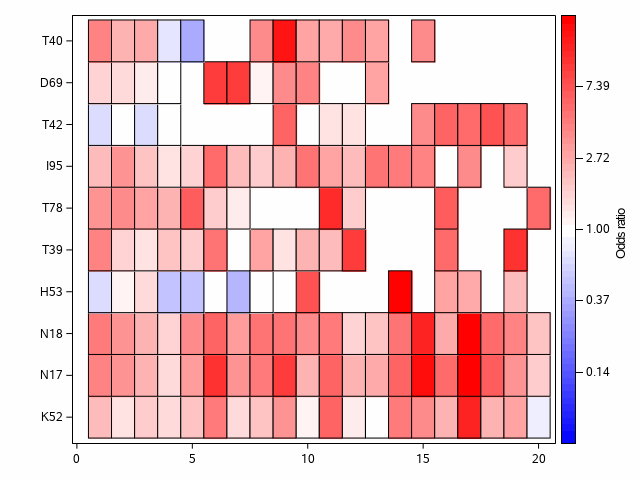 | 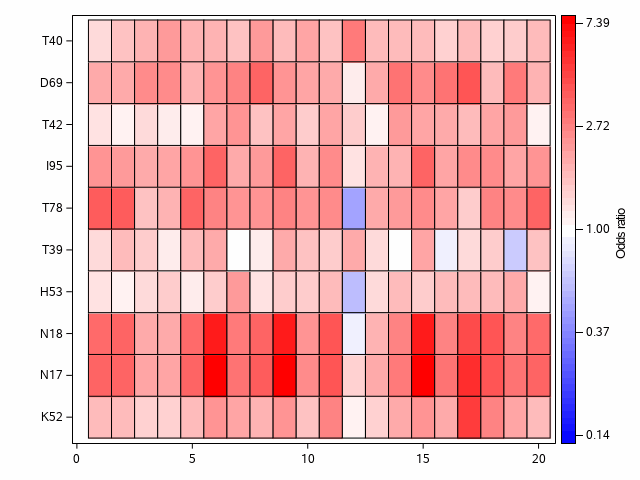 |

**Figure S2: Heatmap of adjusted ORs of emergency hospital admission stratified by ICD10 hospital admission codes (for medication classes with statistically significant ORs concomitant exposure compared to non-use)**

Legends Figure S2

X variables concomitant medication groups

1=cefalexin-aciclovir

2=doxycycline-fluconazole

3=flucloxacillin-fluconazole

4=trimethoprim-naproxen

5=cefalexin-naproxen

6=trimethoprim-ibuprofen

7=cefalexin-ibuprofen

8=erythromycin-fentanyl

9=ciprofloxacin-diclofenac

10=cefalexin-diclofenac

11=trimethoprim-diclofenac

12=erythromycin-citalopram

13=flucloxacillin-paracetamol

14=doxycycline-paracetamol

15=flucloxacillin-atorvastatin

16=doxycycline-atorvastatin

17=erythromycin-atorvastatin

Y variable: ICD10 admission code

S72=Fracture of femur

I63=Cerebral infarction

R55=Syncope and collapse

I21=Acute myocardial infarction

J44=Other chronic obstructive pulmonary disease

I48=Atrial fibrillation and flutter

I50=Heart failure

I20=Angina pectoris

R10=Abdominal and pelvic pain

G45=Transient cerebral ischemic attacks and related syndromes

S82=Fracture of lower leg, including ankle

R29=Other symptoms and signs involving the nervous and musculoskeletal systems

M25=Other joint disorder, not elsewhere classified

K92=Other diseases of digestive system

K80=Cholelithiasis

S52=Fracture of forearm

R04=Haemorrhage from respiratory passages

M79=Other and unspecified soft tissue disorders, not elsewhere classified

N17=Acute kidney failure

M54=Dorsalgia

**Figure S2**


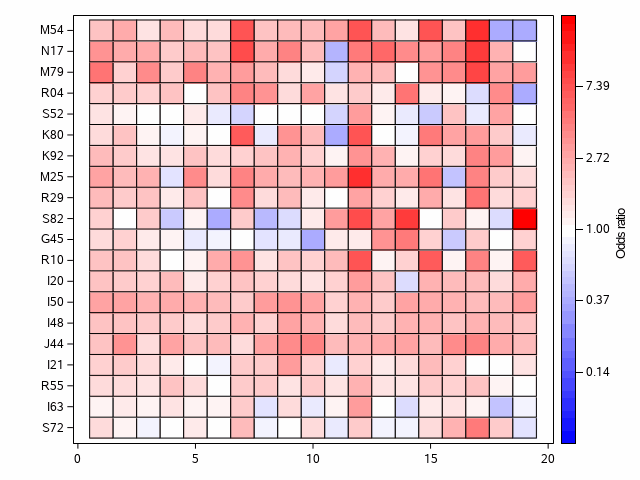

Supplement: Supplementary file 1 — Appendix S1 [file CPT-113-423-s001.docx]
